# Effectiveness of Alberta Family-Integrated Care on Neonatal Outcomes: A Cluster Randomized Controlled Trial

**DOI:** 10.3390/jcm10245871

**Published:** 2021-12-14

**Authors:** Madeleine Murphy, Vibhuti Shah, Karen Benzies

**Affiliations:** 1 Department of Pediatrics, Mount Sinai Hospital, 600 University Avenue, Toronto, ON M5G 1X5, Canada; madeleine.murphy@sickkids.ca (M.M.); vibhuti.shah@sinaihealth.ca (V.S.); 2 Faculty of Nursing, University of Calgary, 2500 University Drive NW, Calgary, AB T2N 1N4, Canada; 3 Departments of Pediatrics and Community Health Sciences, Cumming School of Medicine, University of Calgary, 3300 University Drive NW, Calgary, AB T2N 4N1, Canada

**Keywords:** moderate and late preterm infant, newborn, family-integrated care, neonatal intensive care unit, health services research, family-centred care

## Abstract

Family-Integrated Care (FICare) empowers parents to play an active role as a caregiver for their infant in the neonatal intensive care unit (NICU). This model of care is associated with improved neonatal outcomes, such as improved weight gain and higher breastfeeding rates at discharge in infants admitted to level III NICUs; however, its effectiveness in level II NICUs remains unproven. The objective of this study was to evaluate the effectiveness of the model on neonatal outcomes in a cluster randomized controlled trial conducted in 10 level II NICUs randomized to Alberta FICare or standard care. Mothers and their preterm infants born between 32^+0^ and 34^+6^ weeks’ gestational age were included. The primary outcome was the proportion of infants who regained their birth weight (BW) after 14 days of life. The analysis included 353 infants/308 mothers at Alberta FICare sites and 365 infants/306 mothers at standard care sites. There was no difference in the proportion of infants who had regained their BW by 14 days between the groups. A lack of perceived improved weight gain trajectory for those in the FICare group is attributed to a shorter length of hospital stay and infants being discharged prior to regaining BW.

## 1. Introduction

Globally each year, 15 million infants are born preterm [1]. Of these, 84% [2] are born moderate (32 weeks and zero days to 33 weeks and 6 days gestational age (GA)) or late preterm (34 weeks and zero days to 36 weeks and 6 days GA), with most infants requiring neonatal intensive care. Neonatal intensive care units (NICUs) are stressful environments and parents of admitted infants reported feelings of anxiety, distress, and loss of control [3,4]. One of the major stressors is secondary to an alteration in the parent role and parent–infant separation [5,6]. Early parent–infant interaction in preterm infants was associated with improved breastfeeding rates [7,8], neurodevelopmental outcomes [9,10], and reduced length of stay [8].

Family-Integrated Care (FICare) [11], built on findings reported by Adik Levin [12], was developed as a structured model of care in the NICU that includes parents as integral members of the care team. In an international, multi-centre, cluster randomized controlled trial (RCT) in level III NICUs [13], O’Brien et al. reported that FICare improved infant weight gain, decreased parent stress and anxiety, and increased rate of high-frequency (>6 feedings per day) breastmilk feeding at discharge. The weight gain trajectory over a 21-day period was significantly higher among infants in the FICare group than the standard care group. Similarly, Hei et al. [14] reported improved clinical outcomes and a reduced cost in a cluster RCT of FICare for preterm infants at 11 level III NICUs in China. Improved neonatal outcomes, such as shorter length of stay, shorter duration of supplemental oxygen, reduction in nosocomial infections and antibiotic exposure, and a reduction in rehospitalization rates, were reported for those in the FICare group [14]. Breastfeeding rates and weight gain velocity were significantly higher at discharge for infants in the FICare group compared to those in the standard care group [15]; these differences were maintained when re-evaluated at 18-month follow up. In a pre-post intervention study of FICare, evaluating the clinical outcomes of preterm infants with bronchopulmonary dysplasia (BPD) in two tertiary NICUs in China [16], compared to standard care, the FICare group had significantly increased breastfeeding rates, enteral nutrition time, and weight gain. In addition, infants spent significantly less time on respiratory support.

A different model, Family Nurture Intervention (FNI), was evaluated by Welch et al. [17] in a level IV NICU, where infants were randomized after birth to FNI or standard care. Welch et al. [17] reported no between-group difference in the primary outcome of length of stay. However, there was a trend towards less feeding problems in the NICU [17], more positive mother–infant face-to-face engagement at 4 months [18], and improved social-relatedness, attention, and neurodevelopment of preterm infants at 18-month follow up [19] in the FNI group.

The FICare model was studied predominantly in level III NICUs. In order to address this gap, the Alberta FICare cluster RCT [20] was conducted in 10 level II NICUs that were randomized to Alberta FICare or standard care. The length of hospital stay was significantly shorter for infants in the Alberta FICare group than those in the standard care group, without concomitant increases in emergency department visits or readmissions to 2 months of corrected age [21]. The objective of this study was to report on the effectiveness of the Alberta FICare model on additional neonatal outcomes in level II NICUs with the primary objective being the proportion of infants who regained their birth weight (BW) by 14 days of life.

## 2. Materials and Methods

### 2.1. Study Design and Setting

The sample comprises infants enrolled in the Alberta FICare level II study, a parallel group, superiority, cluster RCT with convenience sampling [20]. This study was conducted across all 10 level II NICUs in the province of Alberta, Canada. Alberta has a single, publicly funded health service delivery system (Alberta Health Services), and many of the structures and processes across hospitals in the province are standardized. Alberta has a population of 4.4 million people [22] with 24% visible minority [23], and around 50,000 births per year [22]. Alberta’s preterm birth rate (9.2%) is the highest among the Canadian provinces [24]. The study was approved by the University of Calgary, Conjoint Health Research Ethics Board (ID 15–0067), University of Alberta, Health Research Ethics Board (Pro00060324), and Covenant Health, Health Research Ethics Board (ID 1762).

### 2.2. Participant Inclusion and Exclusion Criteria

Mothers and their preterm infants born between 32^+0^ and 34^+6^ weeks’ GA with a primary admission, or transfer within 72 h to one of the level II NICUs, were eligible for inclusion. Mothers were included in the intervention group if they agreed to spend a minimum of 6 h per day with their infant. On average, mothers in the Alberta FICare group spent 9 h/day and those in the standard care group spent 7.8 h/day in NICU; the difference was statistically significant [21]. Mothers whose health, social, or language issues inhibited their ability to communicate with the healthcare team were ineligible. Mothers with triplets or higher-order multiple births, and mothers whose infants required palliative care or had severe congenital or chromosomal anomalies were also ineligible.

### 2.3. Randomization and Blinding

The 10 participating level II NICUs were stratified by size with simple random sampling within stratum to ensure balance of intervention (Alberta FICare) or standard care (control) in each stratum. Each group had an equal number of small and large clusters. There are three level II NICUs in each of the two larger cities, and at least one of the hospitals in each city was randomized to the Alberta FICare group. While we did not directly compare urban vs. regional sites, we examined whether group allocation (Alberta FICare vs. standard care) made a difference in each stratum (urban vs. regional sites). Given the risk of contamination in the larger cities due to some nurses and physicians working at more than one site, we asked administrators and healthcare providers in the Alberta FICare group not to discuss the intervention study outcomes with colleagues in the standard care group. The intervention could not be blinded.

### 2.4. Intervention Group: Alberta FICare

Mothers and their infant(s) at each site allocated to the intervention group received Alberta FICare as the routine care. Alberta FICare is a dynamic, psycho-educational intervention with three main components: (1) relational communication, (2) parent education supported by technology and defined learning pathways, and (3) parent support from professionals and family mentors who had previous experience caring for a preterm infant in NICU. A detailed description of Alberta FICare was previously reported [21].

### 2.5. Comparison Group: Standard Care

Mothers and their infant(s) allocated to the standard care group received care as usual. Any changes to policy and practice that may have influenced care at the standard care sites were noted.

### 2.6. Outcome Measures

The primary outcome was the proportion of infants who regained their BW by 14 days of life. Pre-specified secondary outcomes were the following: (1) incidence of apnea of prematurity, (2) duration of total parenteral nutrition (TPN), (3) time to achieve full enteral feeds, (4) time to first skin-to-skin contact, (5) time to first breast feed, (6) time to regain BW, and (7) proportion of infants who had regained BW at discharge.

### 2.7. Statistical Analysis

We chose the primary outcome variable to be the proportion of infants who had regained their BW by 14 days. This was calculated using the following outcome variable: days to regain birth weight. Since this was only measured until discharge, the values for infants who had not regained BW by discharge were right censored at discharge using their length of stay. Like other statistical methods, survival analysis techniques rely on stochastic independence across subjects for valid inference. In our study, some of the observations were clustered at the individual hospital level by twins. To account for this clustering, we used the Nelson–Aalen estimator of the cumulative hazard rather than the Kaplan–Meier estimate of the survival function. The robust variance was calculated using an infinitesimal jackknife variance estimate. The Cumulative Hazard Function *H(t)* and the Survival Function *S(t)* and are linked by the equation:

$$H\left(t\right)=-log_{e}S\left(t\right)$$

We extracted the 14-day value of the cumulative hazard function and its 95% confidence interval (CI) from the output of the analysis for each hospital. These values were then combined using a fixed effects model, which used the inverse variance method weighted according to the number of infants in each hospital. The groups were then compared using a Chi-squared test. Combined estimates with 95% CI were back transformed using the exponential function to yield the survival function. This was used to determine the percentage of infants who had regained their BW by 14 days. For secondary outcomes, dichotomous data were expressed as proportions and compared using Chi-squared tests. Continuous variables were expressed as mean (SD) and compared with a parametric test (i.e., independent *t*-test) when normally distributed; they were expressed as median (IQR) and compared with a non-parametric test (i.e., Mann–Whitney U test) when the distribution was not normal.

## 3. Results

**Figure 1:**
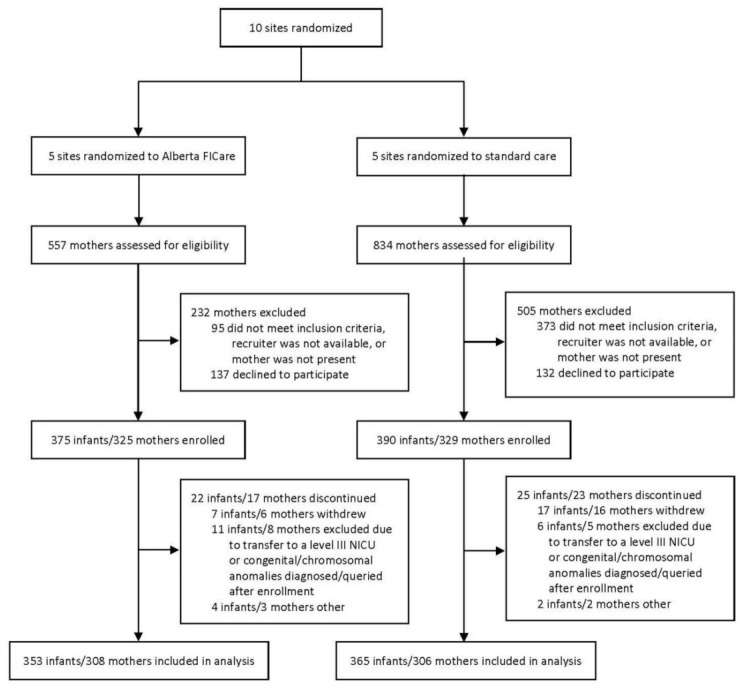
Study flow diagram. Adapted from Benzies et al. [21].

Between December 2015 and July 2018, 765 infants and 654 mothers were enrolled across 10 participating sites: 375 infants/325 mothers at Alberta FICare sites and 390 infants/329 mothers at standard care sites (Figure 1). An equal proportion of infants (6%) in both groups were excluded, most commonly due to becoming ineligible (e.g., transfer to a level III NICU) or their mothers withdrawing from the study. The final sample included 353 infants/308 mothers in the Alberta FICare group and 365 infants/306 mothers in the standard care group. Of the mothers in the Alberta FICare group, 241 (78.2%) were at an urban site and 67 (21.8%) at a regional site. Of those in the standard care group, 272 (88.9%) were in urban sites and 34 (11.1%) at a regional site.

Except that mothers in the Alberta FICare group received antenatal steroids more often and had a lower incidence of twin pregnancy, and their infants more often received a diagnosis of respiratory distress syndrome, mother and infant characteristics at study entry were well matched (Table 1).

There was no difference in the proportion of infants who regained BW by 14 days of life between Alberta FICare and standard care groups (70.4% (95% CI 63.5%, 75.9%) vs. 66.0% (95% CI 59.6%, 71.3%), *p* = 0.381) (Table 2); there was no notable heterogeneity within treatment groups (*p* = 0.015). At the urban sites, there was no difference in the proportion of infants who regained BW by 14 days between Alberta FICare and standard care groups (67.2% (95% CI 46.8, 79.6) vs. 65.8% (95% CI 58.9, 71.6), *p* = 0.771), but there was heterogeneity within the treatment group (*p* = 0.04). At the regional sites, significantly more infants at Alberta FICare sites regained their BW by 14 days compared to those at standard care sites (89.1% (95% CI 78.3, 94.6) vs. 67% (95% CI 46.8, 79.6), *p* = 0.01), with no difference between hospitals within treatment type (*p* = 0.99).

Infants at Alberta FICare sites were first placed skin-to-skin earlier (mean (SD) 0.7 (0.8) vs. 1.1 (1.2) days, *p* < 0.001), achieved full enteral feeds more quickly (mean (SD) 4.1 (3.3) vs. 4.6 (2) days, *p* = 0.03), and spent less time on parenteral nutrition (mean (SD) 4.1 (2.2) vs. 4.7 (2.7) days, *p* = 0.02) compared to those at standard care sites (Table 3). Significantly fewer infants at Alberta FICare sites had regained BW at the time of discharge compared to those at standard care sites (248 infants (70.5%) vs. 283 infants (78%), *p* = 0.02).

## 4. Discussion

In our cluster RCT, conducted with moderate and late preterm infants admitted to a level II NICU in Alberta, there was no difference in the proportion of infants who had regained their BW by 14 days between Alberta FICare or standard care groups. This is in contrast to the study by O’Brien et al. [13] who reported improved infant weight gain and weight gain trajectory over a 21-day period among infants receiving a different model of FICare in level III NICUs. In our study, however, significantly fewer infants in the Alberta FICare group had regained BW by time of discharge compared to those in the standard care group. We previously reported [21] that the length of hospital stay for infants was significantly shorter in the Alberta FICare group compared to the standard care group, and that there was no between group difference in the proportion of infants requiring hospital readmission or emergency department visits until 2 months of corrected age. We speculate that we did not observe the improved weight gain trajectory for those in the Alberta FICare group, as seen in the level III FICare studies [13,14], because we monitored weight over a shorter duration (14 vs. 21 days), and many infants were discharged before they had regained BW. We find it promising that infants at the Alberta FICare sites with a shorter length of stay were more often discharged before regaining BW. Yet, this did not translate into an increased number of hospital attendances following discharge. It appears that infants were discharged when medically ready. Clinically, this is important, since the regain of BW did not appear to be a factor in the decision for readiness for discharge. We know other factors such as maternal confidence play a role in readiness for discharge. At discharge, mothers at the Alberta FICare sites reported slightly higher parenting self-efficacy than mothers at the standard care sites [21]. However, this difference was not statistically significant. Failure to find group differences in parenting self-efficacy is likely because no infant would be discharged before parents are confident in providing care at home.

In the regional hospitals, significantly more infants in the Alberta FICare group had regained their BW by 14 days compared to those in the standard care group. This effect was not observed in those enrolled in the urban hospitals. To our knowledge, regional and urban variation in regaining BW by 14 days is a novel finding.

The impact of early skin-to-skin contact is of great importance. In our study, infants in the Alberta FICare group had significantly earlier skin-to-skin contact than infants in the standard care group. In a meta-analysis of 8 trials with 1736 infants, mainly in resource-limited settings, those who received kangaroo mother care after stabilization had a 40% lower mortality than those who received conventional care [25]. In a recent multicentre RCT of 3211 infants born between 1.0 and 1.799 kg, infants randomized to receive kangaroo mother care before stabilisation had a lower mortality at 28 days compared to those who received conventional care [15]. While it is unlikely that the earlier skin-to-skin care with infants in the Alberta FICare group had any effect on mortality, it is possible that it had some positive effect. In a randomized trial of intermittent early versus late kangaroo mother care, early kangaroo mother care significantly increased exclusive human milk feeding and direct breastfeeding among low-birth-weight infants [26]. Infants in the Alberta FICare group spent less time on TPN, potentially avoiding exposure to TPN-related complications and associated costs. Infants in the Alberta FICare group also achieved full enteral feeds earlier, which may have an impact on readiness for discharge. For outcomes relating to time, the results needed to be interpreted with caution, because there was variation in how sites documented day of life, which we recognize as a limitation of the study.

Agreement to participate in the study at the Alberta FICare sites required that a parent of the infant agreed to spend 6 h per day with their infant in NICU. If a potential participant did not agree to spend 6 h per day in the NICU, they were deemed ineligible. We were unable to determine how many mothers were ineligible and could not spend 6 h per day in the NICU because this information was collected inconsistently, which is a limitation of the study. On average, mothers in the Alberta FICare group spent 9 h/day and those in the standard care group spent 7.8 h/day in NICU. The promising level of parental presence in the standard group may explain the modest intervention effects. This might be a more significant difference to earlier studies, despite not being level III centres.

Family-centred care (FCC) approaches were widely applied in NICUs in an effort to alleviate parental feelings of anxiety and a lack of perceived control and are shown to be associated with a reduced length of stay and risk of moderate-to-severe BPD [27,28]. However, implementation is variable among NICUs [29]. Hei et al. [14] adapted the FICare model that was originally developed in Canada for China, with one of the largest changes being parental presence in the NICU; the prevailing culture in China was that parents were not permitted to be in the NICU and had minimal communication with staff. The Alberta FICare model is a structured model of care which builds on FCC approaches and its structure lends itself to sustainability. One of the three main components of the Alberta FICare model is relational communication, which includes using circular pattern diagrams, questioning, and commendations as evidence-based strategies [30,31] to facilitate implementation into units. Yet, parental presence and improved communication may not fully explain the effects of FCC on infant and parent outcomes. In a prospective study in 11 neonatal units across 6 European countries, Aija et al. [32] reported that only a few background characteristics of infants and parents explained the differences in parental presence in medical rounds, suggesting that unit culture plays a major role in inviting parents to participate. Future studies should assess unit culture as part of the pre-implementation readiness to implement FICare.

It is increasingly important to implement effective models of care, especially in the post-COVID healthcare system. Infants in the Alberta FICare group spent less time on TPN, were discharged home earlier [21], and this shorter stay did not result in downstream healthcare utilization, all of which may reduce healthcare system costs. A strength of our study is that we had a large sample size with complete data.

## 5. Conclusions

There was no difference in the proportion of infants who had regained their BW by 14 days between groups. A lack of perceived improved weight gain trajectory for those in the Alberta FICare group may be due to a shorter length of hospital stay and infants being discharged prior to regaining BW.

## Figures and Tables

**Table 1:** Baseline maternal and neonatal characteristics of the study population.

Variable	*n*	Alberta FICare	*n*	Standard Care
Maternal characteristics		N = 308		N = 306
Maternal age (years), mean (SD)	303	30.7 (5.7)	295	31.3 (5.3)
Parity, median (IQR)	307	0 (0, 1)	305	0 (0, 1)
Diabetes, *n* (%)	299	52 (17.4)	301	56 (18.6)
Hypertension, *n* (%)	305	53 (17.4)	298	53 (17.8)
Maternal substance use, *n* (%)	305	41 (13.4)	298	38 (12.8)
Antenatal steroids, *n* (%)	291	177 (60.8)	299	157 (52.5)
Cesarean delivery, *n* (%)	308	150 (48.7)	306	143 (46.7)
Twins, n (%)	308	45 (14.6)	306	59 (19.3)
Infant characteristics		N = 353		N = 365
Gestational age (weeks), mean (SD)	353	33.7 (0.83)	365	33.8 (0.81)
Birth weight (grams), mean (SD)	353	2163 (395)	365	2120 (411)
Male sex, *n* (%)	353	190 (53.8)	365	195 (53.4)
Apgar score at 5 min, median (IQR)	351	9 (8, 9)	364	9 (8, 9)
Respiratory distress syndrome, *n* (%)	353	158 (44.8)	364	127 (34.9)
Need for invasive ventilation, *n* (%)	353	16 (4.5)	364	18 (4.9)
Need for non-invasive ventilation, *n* (%)	353	165 (46.7)	364	166 (45.6)

**Table 2:** Primary outcome.

Variable	Alberta FI Care (N = 353)	Standard Care (N = 365)	*p* Value
Regained birth weight by 14 days			
Group overall	70.4% (63.5, 75.9)	66% (59.6, 71.3)	0.381
Urban hospitals	67.2% (46.8, 79.6)	65.8% (58.9, 71.6)	0.771
Regional hospitals	89.1% (78.3, 94.6)	67% (46.8, 79.6)	0.01

**Table 3:** Secondary outcomes.

Variable	*n*	Alberta FICare (N = 353)	*n*	Standard Care (N = 365)	*p* Value
Apnea of prematurity, *n* (%)	351	166 (47.3)	349	164 (47.0)	0.94
Duration of parenteral nutrition, days, mean (SD)	154	4.1 (2.2)	179	4.7 (2.7)	0.02
Time to full enteral feeds, days, mean (SD)	342	4.1 (3.3)	336	4.6 (2.0)	0.03
Time to first skin-to-skin, days, mean (SD)	329	0.7 (0.8)	313	1.1 (1.2)	<0.001
Time infant first put to breast, days, mean (SD)	319	2.6 (3.5)	327	2.8 (3.5)	0.48
Time to regain birth weight, days, mean (SD)	247	11.4 (3.5)	272	11.5 (3.7)	0.86
Regain birth weight at discharge, *n* (%)	352	248 (70.5)	363	283 (78)	0.02

## Data Availability

The data presented in this study are available on request from the corresponding author.
